# Molecularly Imprinted Polymer-Based Electrochemical Sensor for the Detection of Azoxystrobin in Aqueous Media

**DOI:** 10.3390/polym16101394

**Published:** 2024-05-14

**Authors:** Vu Bao Chau Nguyen, Jekaterina Reut, Jörg Rappich, Karsten Hinrichs, Vitali Syritski

**Affiliations:** 1 Department of Materials and Environmental Technology, Tallinn University of Technology, Ehitajate tee 5, 19086 Tallinn, Estonia; vunguy@taltech.ee (V.B.C.N.);; 2 Young Investigator Group Nanoscale Solid-Liquid Interfaces, Helmholtz-Zentrum Berlin für Materialien und Energie GmbH, Schwarzschildstr. 8, 12489 Berlin, Germany; rappich@helmholtz-berlin.de; 3 Application Laboratories Berlin, Leibniz-Institut für Analytische Wissenschaften-ISAS-e.V., Schwarzschildstraße 8, 12489 Berlin, Germany; hinrichs@isas.de

**Keywords:** molecularly imprinted polymer, environmental pollutant, fungicide, azoxystrobin, electrochemical sensor

## Abstract

This work presents an electrochemical sensor detecting a fungicide-azoxystrobin (AZO) in aqueous environments. This AZO sensor utilizes a thin-film metal electrode (TFME) combined with an AZO-selective molecularly imprinted polymer (AZO–MIP). The AZO–MIP was directly generated on TFME through electrochemical polymerization from the solution containing two functional monomers: aniline (Ani) and m-phenylenediamine (mPD), and the template: AZO, which was afterwards removed to form AZO-selective cavities in the polymer matrix. The AZO–MIP preparation was characterized by electrochemical and ellipsometry measurements. Optimization of the synthesis parameters, including the charge density applied during electrodeposition, the monomer-to-template ratio, was performed to enhance the sensor’s performance. The results demonstrated that the AZO sensor achieved a low limit of detection (LOD) of 3.6 nM and a limit of quantification (LOQ) of 11.8 nM in tap water, indicating its sensitivity in a complex aqueous environment. The sensor also exhibited satisfactory selectivity for AZO in both ultrapure and tap-water samples and achieved a good recovery (94–119%) for the target analyte. This study highlights the potential of MIP-based electrochemical sensors for the rapid and accurate detection of fungicide contaminants in water, contributing to the advancement of analytical tools for water-quality monitoring and risk assessment.

## 1. Introduction

The presence of various organic pollutants in environmental water poses significant risks to both human and environmental health. Agrochemicals, namely fungicides, have garnered significant interest among these pollutants. This is because they are essential for ensuring food security, which leads to an increase in their preventive use. However, this rise in consumption comes with a significant level of toxicity to a broad range of organisms and aquatic biota [1]. Azoxystrobin (AZO), a common fungicide used in agriculture, belongs to the strobilurin class and is renowned for its broad-spectrum effectiveness against a wide range of fungal pathogens [2]. It functions by suppressing the respiratory chain in fungi, therefore impeding their growth and reproduction [3].

According to a recent report from Maximize Market Research, the global AZO market exhibited a substantial growth trajectory, escalating from USD 0.91 billion in 2021 to a remarkable projection of USD 1.93 billion by 2029 [4]. This remarkable surge in AZO usage has triggered profound concerns about its environmental impact, particularly concerning potential waterbody contamination [5]. For instance, the presence of AZO contamination has been detected in freshwater ecosystems in South Africa and China, alongside other organic pollutants [6,7]. One major concern associated with AZO is its significant threat to aquatic ecosystems due to its highly toxic nature. According to the European Food Safety Authority, AZO can rapidly eradicate up to 50% of aquatic organisms within a short period, even at concentrations as low as 1 mgL^−1^ [8]. This alarming toxicity level is a cause for concern, especially when considering its adverse effects on fishes, particularly those where exposure to the pollutant can cause them to suffer from various health issues, including physiological disruptions, reduced growth rates, reproductive disorders, and even mortality [9,10,11,12]. Studies have demonstrated that AZO causes oxidative stress and apoptosis in aquatic species, impacting their mitochondrial respiration. [11]. Moreover, prolonged exposure to AZO could disturb the fragile equilibrium of aquatic ecosystems. For example, AZO concentrations in the range of 0.2–0.5 mgL^−1^ significantly reduce the growth of eukaryotic algae in freshwater [13], and they can harm aquatic plants by inhibiting antioxidant enzymes and causing DNA damage [14].

Furthermore, the potential risks of AZO exposure extend to human health by contaminating water supplies. Extended exposure to AZO has been associated with suppression of mitochondrial oxidative respiration and changes in neuronal cell lipid abundance [15]. Also, genotoxic activity risk has been identified, showing varying effects with short-term and long-term exposure, depending on age groups [16].

Conventional analytical techniques, including high-performance liquid chromatography (HPLC) [17,18] and gas chromatography-mass spectrometry (GC-MS) [19] have been widely used for analyzing azoxystrobin. However, these techniques often require expensive equipment and skilled personnel, rendering them impractical for quick on-site evaluations. Given the widespread use and persistence of AZO in water bodies, the development of simple and commercially feasible sensing technologies to detect AZO becomes imperative to assess and mitigate the associated risks.

In designing chemo/biosensors, molecular imprinting technique stands out as an indispensable means of generating the state-of-the-art recognition elements called molecularly imprinted polymers (MIPs). MIPs, crosslinked polymer matrices with engraved molecular recognition sites specific for the target analyte [20], have distinguished themselves as versatile biomimetic receptors in analytical chemistry, offering promising alternatives to biological receptors for sensitive and selective target analyte detection [21]. MIPs integrated with various sensor transducers have been extensively studied for the detection of numerous environmental pollutants [22,23]. Electrochemical sensors, in particular, have garnered significant interest. They offer real-time monitoring of analytes, simplicity in handling, and sufficient sensitivity at a reduced cost. Additionally, their compatibility with microfluidic systems, large-scale fabrication, and multiplexing technologies makes them an ideal starting point for realizing low-cost and portable sensing platforms suitable for the in-field detection and quantification of environmental contaminants [24,25].

Despite the potential of MIP-based electrochemical sensors, their development for AZO detection in water remains limited. To the best of our knowledge, the synthesis of MIPs tailored for AZO recognition and their subsequent application in electrochemical sensing platforms have not been previously reported.

This work aims to address this knowledge gap by developing a MIP-based electrochemical sensor for the sensitive and selective detection of AZO in aqueous media. Two functional monomers, namely, aniline (Ani) and meta-phenylenediamine (mPD), were selected among other electropolymerizable monomers through computer modelling. This involved estimating the geometric optimization using Universal Force Field (UFF) and the binding energies using density functional theory (DFT)–based quantum chemical calculation. The successful implementation of this AZO–MIP-based sensor (AZO sensor) will provide a valuable tool for the rapid on-site monitoring of AZO in water, facilitating early detection and mitigation of potential risks associated with its presence.

## 2. Materials and Methods

### 2.1. Chemicals and Materials

All used chemicals, except acetic acid supplied by Lach-ner, were purchased from Sigma, St. Louis, MO, USA. The chemicals were received and stored under standard conditions. The aqueous solutions were prepared with ultrapure water (Direct-Q 3 UV Water Purification system, resistivity 18.2 MΩ cm at 25 °C, Merck KGaA, Darmstadt, Germany). The polymer synthesis was conducted in phosphate buffered saline (PBS) (0.01 M, pH 7.4) containing 20% of acetonitrile. Analyte solutions were meticulously prepared using ultrapure as well as tap water. All electrochemical measurements were conducted in a 0.3 M KCl solution containing 4 mM redox probe K_3_[Fe(CN)_6_]/K_4_[Fe(CN)_6_]. Thin-film metal electrodes (TFME) were obtained from MicruX Technologies, Gijón, Spain, and consisted of a 1 mm diameter circular (approximately 0.785 mm^2^) gold working electrode (WE), a gold reference electrode (RE), and a gold counter electrode (CE). To perform all electrochemical measurements, TFME were connected to an electrochemical workstation (Reference 600, Gamry Instruments, Warminster, PA, USA). The potentials associated with the TFME activation process were referenced against the Ag/AgCl/3 M KCl external reference electrode, while all other potentials were referenced against the RE of TFME.

### 2.2. Functional Monomer Selection

To select the suitable functional monomer, electropolymerizable monomers including 2-methyl-4-nitroaniline (2M4N), 3-aminothiophenol (3ATP), aniline (Ani), meta-phenylenediamine (mPD), pyrazole (PRZ), and pyrrole (PYR) were selected. The binding energy between each monomer and AZO was estimated with a 1:1 ratio. The initial structures of their pre-polymerization complexes were prepared and subjected to geometric optimization using Avogadro 1.2.0 software, employing the Universal Force Field (UFF) for energy minimization. Subsequently, binding energies were computed utilising density functional theory (DFT) method at B3LYP/6-31+G level, employing Gaussian ’09 software packages.

### 2.3. Sensor Preparation

The AZO sensor was prepared by the electrodeposition of an AZO–MIP film on the WE surface of gold TFME. Prior to electrodeposition, the WE and RE of TFME were activated by cycling the potential between 0.5 and 1.55 V in a 0.05 M sulfuric acid solution, with a scan rate of 100 mV/s, for a minimum of 10 cycles. Subsequently, they were rinsed with ultrapure water and dried under a nitrogen atmosphere.

Co-polymer of Ani and mPD film containing AZO, denoted as poly(Ani-co-mPD)/AZO, was synthesized at the constant current of 0.3 µA in a setup where the electrodes of TFME were exposed to a PBS with 20% of acetonitrile containing Ani, mPD and AZO, at the determined optimal concentrations. Molecular imprints of AZO were generated within poly(Ani-co-mPD)/AZO by immersing the modified electrode in a 5% acetic acid solution on vortex for 30 min, followed by ultrapure water for 30 min to remove AZO, resulting in AZO–MIP formation.

To ensure the coexistence of Ani and mPD in the poly(Ani-co-mPD), and to evaluate the efficiency of the removal process using a 5% acetic acid solution, a custom build dry-air purged IR spectroscopic ellipsometer externally attached to a FT-IR spectrometer (Vertex 70, Bruker, Billerica, MA, USA) was employed. The tanΨ measurements were taken in the mid infrared spectral range at incidence angles of 70° and 80°. Additional information regarding the configuration and procedure can be found at [26,27].

The sensor preparation stages were characterized by cyclic voltammetry (CV) and electrochemical impedance spectroscopy (EIS) in a 0.3 M KCl solution containing 4 mM redox probe K_3_[Fe(CN)_6_]/K_4_[Fe(CN)_6_]. CV was performed by cycling the potential between −0.2 and 0.2 V at a scan rate of 50 mV/s while EIS measurements were carried out at an alternating potential with an amplitude 10 mV and a frequency range of 0.1 Hz to 10 kHz.

### 2.4. Evaluation of Sensor Performance

The sensor senses the changes in charge transfer between AZO–MIP-modified WE of TFME and redox probe ions ([Fe(CN)_6_]^3−^/[Fe(CN)_6_]^4−^) before and after incubation in a sample solution containing the target analyte, AZO. Following incubation of the sensor in a sample solution, AZO binds AZO–MIP and hinders the charge transfer to WE of TFME.

Differential pulse voltammetry (DPV) was used to measure this effect and conducted in a 0.3 M KCl solution containing 4 mM redox probe (K_3_[Fe(CN)_6_]/K_4_[Fe(CN)_6_]) at a potential range of 0 to 0.4 V, pulse amplitude of 35 mV, a pulse width of 0.04 s, a sample period of 0.5 s and a step potential of 7 mV (the optimization details of DPV parameters in Section S1).

Thus, the concentration-dependent sensor response, I_n_, can be represented as a normalized-suppression of the DPV current peak as follows:

I_n_ = (I_0_ − I)/I_0_ (1)

where I_0_ and I are the current peak values measured by DPV after incubation in ultrapure water without and with the target analyte, respectively.

All analyte solutions were prepared in ultrapure as well as tap water except otherwise stated in which case the pH was adjusted to the desired value using drops of 1 M HCl or 1 M NaOH.

The limits of detection (LOD) and quantification (LOQ) were determined from the linear regression of the sensor’s response to low concentrations of the analyte in PBS, using Equations (2) and (3), as follows:

LOD = 3SD/b(2)

LOQ = 10SD/b(3)

where SD and b are the standard deviation of the residual and the slope of the regression line, respectively.

The selectivity of the AZO sensor was evaluated by comparing the sensor’s response upon rebinding of AZO and other interfering fungicides, including pyraclostrobin (PRC), kresoxim-methyl (KSX) at the same concentrations in ultrapure water. Similar experiments were conducted in tap water spiked with the desired analyte concentrations. For this purpose, tap-water samples were collected from the water-supply line of Tallinn University of Technology and used without further treatment.

## 3. Results and Discussion

### 3.1. Functional Monomers Selection

**Figure 1:**
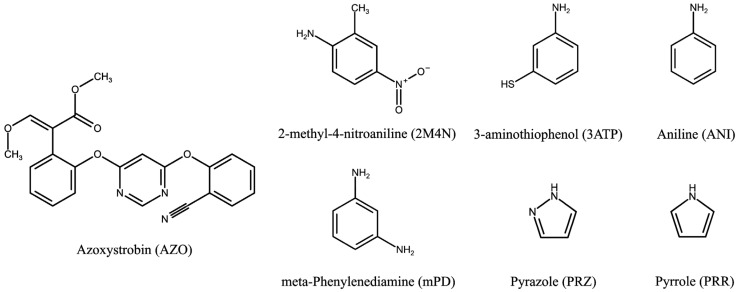
Structural formula of Azoxystrobin (AZO) and electropolymerizable monomers examined as potential functional monomers for AZO–MIP synthesis.

The choice of functional monomer is a critical factor in achieving the optimal performance of a MIP. The effectiveness of MIPs primarily depends on the strength of the interaction between the template molecule and the functional monomer [28]. This interaction facilitates the formation of molecular memory within the MIP by considering both the shape and arrangement of functional groups in the template molecule. The following electropolymerizable monomers with functional groups capable of engaging in hydrogen bonds with AZO’s oxygen atoms were examined: 2M4N, 3ATP, Ani, mPD, PRZ, and PYR (Figure 1). A computational modelling was utilized to compare the binding energies between the monomers and AZO to identify a more stable complex (Table S1). As can be seen, Ani showed the highest binding energies with AZO, signifying its potential to form robust non-covalent interactions (hydrogen bonding) with the template. However, Ani possesses only one amino group, which is capable of hydrogen bonding with AZO and is also involved in the formation of the polymer chain during polymerization. This could potentially compromise the recognition capabilities of the resulting MIP. To address this concern, mPD was introduced as a co-monomer to ensure the hydrogen bonding with AZO, which, along with the contribution of π-π interactions between AZO and both monomers, resulted in enhanced selectivity and stability of the polymer structure. The feasibility of the formation of a co-polymer of Ani and mPD during electrochemical polymerization has been successfully demonstrated in previous studies [29,30]. Additionally, it was found that phenylenediamines can act as branching or crosslinking sites when they undergo copolymerization with Ani [31,32,33]. Hence, by incorporating mPD into the functional monomer mixture, it was expected that a more stable MIP structure with enhanced molecular recognition capabilities would be achieved.

Furthermore, it should be noted that the possible non-covalent interactions between AZO and monomers, mPD and Ani, include, in addition to hydrogen bonding, π-π interactions between the aromatic moieties present in the studied molecules. These interactions can contribute significantly to the strength of non-covalent interaction in the prepolymerization complex as well as in the resulting MIP. Therefore, the composition of the polymerization solution including the use of single or dual monomers as well as the ratio of monomers to the template was optimized experimentally (see Section 3.2 and Figure S5a in Supplementary Information).

### 3.2. Synthesis and Characterization of AZO–MIP Film

In electrochemical MIP synthesis, it is essential to preserve the structural integrity of the template molecules during electropolymerization at an applied potential. Therefore, a preliminary test was conducted to confirm that the AZO remained unoxidized at the electrodeposition potential of the selected monomer.

CV measurements (Figure S2) were performed to determine the appropriate electrochemical potential for synthesis of the co-polymer poly(Ani-co-mPD) while preventing the undesired oxidation of AZO. The CV scans revealed that Ani undergoes oxidation at approximately 300 mV, while mPD oxidizes at around 250 mV. Remarkably, the proximity of Ani’s oxidation potential to that of mPD, suggesting the feasibility of generating a co-polymer using these two monomers due to the potential combination of their electrochemically generated radical cations, enabling the propagation of chain growth. Consequently, it is expected that the oxidation of both monomers in their solution mixture will occur at a potential higher than 300 mV.

In this study, the galvanostatic method was employed for AZO–MIP film synthesis. This method was chosen for its ability to precisely control polymerization through current regulation, enhancing the reproducibility and uniformity of the resulting AZO–MIP films. The current was adjusted to attain the required electrodeposition potential of above 300 mV. By adjusting the time of the galvanostatic process, the polymer film thickness can be precisely controlled, providing tunability in the final MIP film efficiency.

The electrochemical polymerization in this study was performed under neutral pH conditions. In these conditions, Ani molecules easily undergo oxidation, resulting in the formation of a stable non-conductive emeraldine base [33,34,35]. Furthermore, the poly(m-phenylenediamine), or poly(mPD), film synthesized under neutral pH conditions exhibits excellent stability coupled with imprinting capability, making it an ideal choice for MIP layer formation [36,37,38].

**Figure 2:**
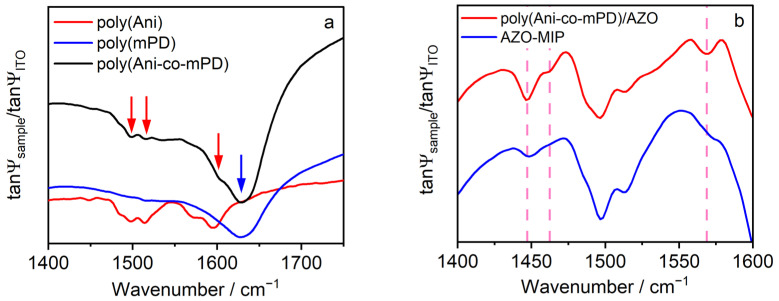
(**a**) Referenced IRSE spectra of poly(Ani), poly(mPD), and poly(Ani-co-mPD) films on ITO; and (**b**) referenced IRSE spectra of poly(Ani-co-mPD)/AZO films on ITO before washing (solid red line) and after washing (solid blue line). Vertical dashed lines show varied responses post-washing process, including disappearance or attenuation.

The electrodeposited thin co-polymer film was characterized using IRSE (Figure 2a). The spectra of polyaniline (poly(Ani)) and poly(mPD) electrodeposited under similar conditions as those of the poly(Ani-co-mPD) film, were presented for comparison. Examining the region of 1400–1700 cm^−1^ of spectra, several characteristic bands can be clearly distinguished. Specifically, the absorption bands in poly(Ani) spectrum at 1498 and 1515 cm^−1^ are attributed to benzenoid (aromatic) ring stretching, while the band at 1595 cm^−1^ with a shoulder at 1570 cm^−1^ is attributed to C=C stretching in a quinoid ring. This may indicate the partially oxidized (emeraldine) form of poly(Ani) [39]. However, in the spectrum of poly(mPD), only one broad absorption band near 1630 cm^−1^ is present, which can be attributed to the stretching mode of quinoid imine [40,41] as well as to the C=C stretching vibration in a phenazine-like segment [40,42]. The spectrum of poly(Ani-co-mPD) now displays the characteristic bands of both poly(Ani) (red arrows) and poly(mPD) (blue arrow) at wavenumbers of 1498, 1515, and 1630 cm^−1^, with a shoulder at 1600 cm^−1^. This suggests that the synthesized co-polymer structure differs from the corresponding homopolymers structures and obviously contains the aromatic rings in combination with quinoid rings as well as phenazine-like segments.

To prepare AZO–MIP, the polymer film synthesized in the presence of AZO (poly(Ani-co-mPD)/AZO) was treated with 5% acetic acid to remove AZO from the polymer matrix. To confirm the removal of AZO, the film before and after the treatment was evaluated using IRSE (Figure 2b). As can be seen, there are changes in the frequency positions in the range 1400–1600 cm^−1^. Specifically, the peak at 1447 cm^−1^ was attenuated, and peaks at 1463 and 1569 cm^−1^ vanished after washing. Since these peak positions are close to those of AZO (Figure S3), it can be concluded that the AZO present in the poly(Ani-co-mPD)/AZO was removed after treatment in an acetic acid solution, presumably resulting in the formation of the AZO–MIP film.

In addition, the formation of AZO–MIP on the Au WE was evaluated by electrochemical techniques, including CV and EIS. These measurements helped to establish a connection between each stage of modification by monitoring alterations in charge transfer between the redox pair in solution and on the Au WE surface. CV analysis indicated a reduction in both anodic and cathodic current peaks following poly(Ani-co-mPD)/AZO electrodeposition (Figure S4a), signifying the development of a non-conductive film hindering charge transfer at the electrode–solution interface. The observed recovery of current peaks after acetic acid treatment suggested improved permeability of the polymer layer due to the formation of imprinted cavities of AZO–MIP. Similar trends were observed in the EIS spectra (Figure S4b), where the semicircle diameter representing charge transfer resistance, Rct, increased after polymer film electrodeposition (Table S2), and then decreased after subsequent treatment with acetic acid to form AZO–MIP. The selective adsorption of AZO by AZO–MIP resulted in changes observed in both the CVs and the EIS spectra.

The advantage of poly(Ani-co-mPD) over the homopolymers poly(Ani) or poly(mPD) for AZO–MIP formation was unequivocally demonstrated by comparing the response signals of the sensors equipped with corresponding MIPs for AZO detection (Figure S5a). As shown, the sensor with the MIP synthesized from the co-polymer exhibits a superior response compared to the sensors with the MIPs prepared from poly(Ani) or poly(mPD). In addition, the MIP-based sensor synthesized using co-monomers also shows better performance in comparation with its reference non-imprinted polymer (NIP)-based sensor (Figure S5b), indicating the effectiveness of molecular imprinting.

#### 3.2.1. Effect of Thickness

**Figure 3:**
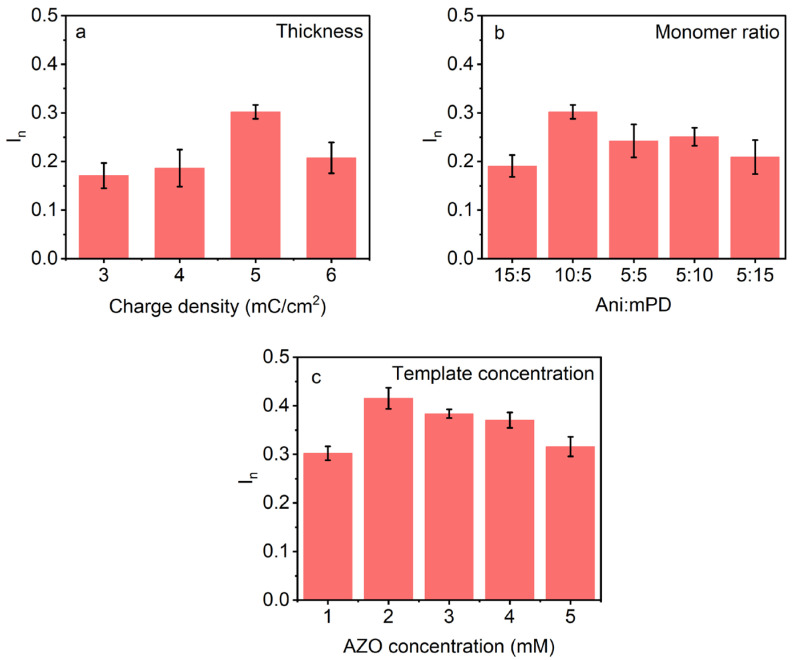
Optimization of AZO–MIP preparation. Responses of the sensors equipped with various AZO–MIPs after incubation in 10 µM AZO in ultrapure water. AZO–MIPs were prepared using different: (**a**) charge densities; (**b**) Ani:mPD ratios; and (**c**) AZO concentrations at constant Ani:mPD ratio of 10:5.

To determine the optimal polymer thickness of the AZO–MIP, at which the sensor produces the highest response towards AZO, the WE of TFME was modified with poly(Ani-co-mPD)/AZO films of various thicknesses generated by passing different charge densities, ranging from 3 to 6 mC/cm^2^. As can be seen, the sensor equipped with AZO–MIP formed from film generated by 5 mC/cm^2^ demonstrates the highest response and was consequently selected as optimal for further study (Figure 3a).

#### 3.2.2. Effect of the Monomer to Template Ratio

The recognition capabilities of an MIP can be influenced by the template:monomer ratio used to build the MIP [36]. A high amount of monomer may reduce binding selectivity due to increased non-specific binding, while a low amount of monomer may decrease the number of binding sites in the formed polymer due to insufficient interactions with the template [43]. Therefore, the optimal Ani:mPD and AZO:Ani-co-mPD molar ratios were determined. As can be seen, the sensor equipped with AZO–MIP prepared from the synthesis solution containing 10 mM of Ani and 5 mM of mPD exhibits the most favorable response towards AZO (Figure 3b). Furthermore, by keeping these concentrations in the synthesis solution constant and varying the concentrations of AZO from 1 to 5 mM, it was determined that the presence of 2 mM of AZO resulted in the highest responsiveness of the AZO sensor (Figure 3c). Thus, the optimal concentration ratio of AZO:Ani:mPD in the synthesis solution was determined to be 2:10:5 mM and was used for further experimental investigations.

### 3.3. Performance of AZO Sensor

#### 3.3.1. Effect of pH

**Figure 4:**
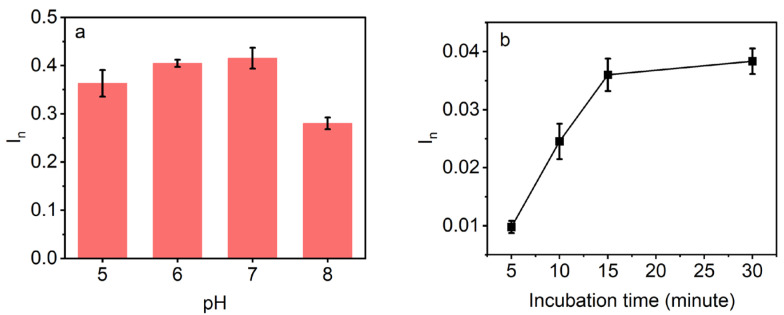
(**a**) Responses of AZO sensor upon incubation in 10 µM AZO solution in ultrapure water of different pH values; and (**b**) effects of incubation times on the DPV responses of AZO sensor after rebinding in 50 nM AZO solution in ultrapure water.

The influence of the pH of the analyzed solution on the sensor’s response was studied to ensure accurate and reliable detection of AZO in real-world aqueous environments. The responses of AZO sensor were evaluated upon incubation in 10 µM AZO aqueous solution at pH values ranging from 5 to 8, aligning with the natural pH levels found in environmental water [44,45,46,47]. Hydrolysis and decomposition of AZO molecules can occur at lower pH values, while higher pH values may cause degradation due to nucleophilic substitution reactions [48]. The data presented in Figure 4a demonstrated an ascending trend in sensor response with increasing pH until 7, followed by a subsequent lowering of the signal.

#### 3.3.2. Effect of Incubation Time

A rapid response time is a desirable characteristic for chemical sensors in environmental monitoring. In this study, the total time required to obtain the detection result includes the electrochemical DPV measurements, which take a maximum of 5 min, and sensor incubation in an analyte solution to allow for its rebinding on MIP, which may require a longer amount of time. Hence, the optimal incubation time for the AZO sensor was experimentally determined by measuring the sensor’s responses upon incubating in the 50 nM AZO solution for various durations, including 5, 10, 15, and 30 min (Figure 4b). The findings indicate that the sensor’s response substantially increases as the incubation time is prolonged up to 15 min. Beyond this point, any further increase is marginal. Considering the response signal values and the plateau effect observed beyond 15 min, this incubation time was deemed sufficient to achieve optimal analyte binding to the AZO–MIP.

#### 3.3.3. Sensitivity Study

**Figure 5:**
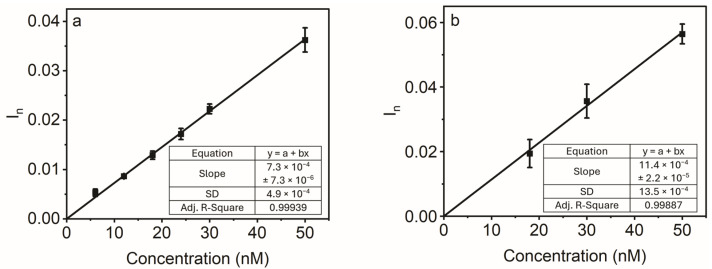
Performance of the AZO sensor at low analyte concentrations (6–50 nM) in: (**a**) ultrapure water; and (**b**) tap water. The solid line is a linear regression fit. The error bars represent standard deviation of three measurements carried out by three independent AZO sensors.

The purpose of the sensitivity study was to evaluate the performance of the AZO sensor in detecting low analyte concentrations and to determine LOD and LOQ. As shown in Figure 5, the response of the AZO sensor exhibited a quasi-linear increase in response signal with rising analyte concentrations in the range of 6–50 nM. In the case of AZO spiked into ultrapure water (Figure 5a), the sensor could detect it with LOD and LOQ of 2.0 nM and 6.7 nM, respectively. However, when the sensor was tested in AZO solutions prepared in tap water (Figure 5b), its responses exhibited higher variations, as indicated by an elevated standard deviation, and subsequently raised the LOD and LOQ values of 3.6 nM and 11.8 nM, respectively. This could be attributed to tap water, which predominantly contains multiple dissolved ions, minerals, and other compounds [49,50,51], thereby leading to an increased background signal and noise. Nevertheless, AZO concentrations in the lowland stream water were found to be as high as 73.6 nM [52], indicating the practical utility of the AZO sensor for the analysis of environmental water.

#### 3.3.4. Selectivity Study

**Figure 6:**
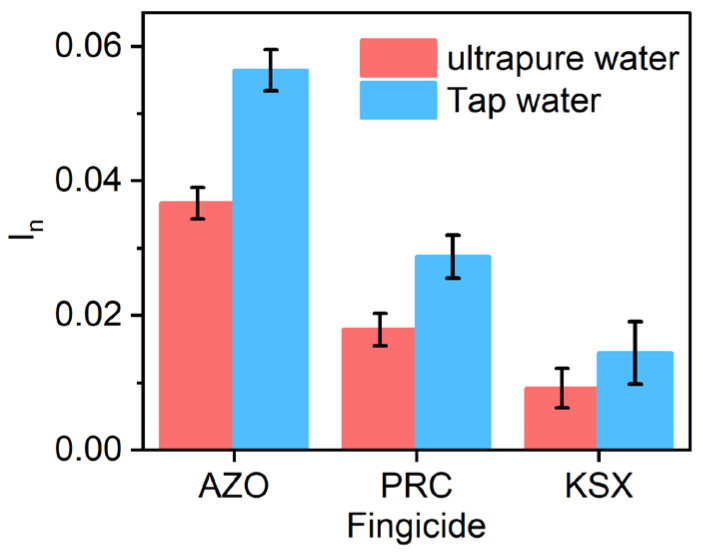
Responses of the AZO sensor after incubating in a 50 nM concentration of each different fungicide (AZO, PRC, and KSX) in ultrapure and tap water.

Two fungicides, kresoxim-methyl (KSX) and pyraclostrobin (PRC), which belong to the same group of strobilurin fungicides as AZO, were chosen for evaluating the sensor’s ability to distinguish between different compounds (refer to Figure S6 for detailed structures). Despite their structural similarities, the AZO sensor exhibits a stronger response to the target compound (AZO) compared to the other tested fungicides (KSX and PRC) (Figure 6). In general, the signal caused by the AZO presence was approximately two and four times higher than the signals generated by PRC and KSX, respectively (Table S3). Hence, the AZO sensor is capable of discriminating between AZO and other molecules in the tap-water matrix.

To validate the accuracy of the sensor’s measurements and assess its practical utility, a series of recovery experiments were conducted using AZO solutions of varying concentrations in tap-water (as detailed in Table 1). The sensor showed good recoveries within the range of 94% to 119%, confirming its robust performance.

## 4. Conclusions

This report showcases the successful development of an electrochemical sensor with a robust MIP-based sensing element for detecting the fungicide AZO in water. The sensor combines the selectivity of AZO–MIP with the low-cost and compact TFME. To enhance the selectivity of AZO–MIP, it was synthesized by co-polymerization of two functional monomers, Ani and mPD. Following optimization, the sensor exhibited appreciable recognition of AZO and was capable of detecting low levels of analyte with an LOD value of 3.6 nM and an LOQ value of 11.8 nM in tap water. Importantly, the sensor showcased good selectivity, effectively distinguishing AZO from similar fungicide compounds (PRC and KSX) both in ultrapure and tap-water samples. Additionally, the sensor demonstrated a good recovery, ranging from 94% to 119% in tap-water samples. Considering that AZO concentrations can be found in the lowland stream water that are as high as 73.6 nM, the practical utility of the AZO sensor for the cost-effective on-site detection of AZO in environmental water is evident.

## Figures and Tables

**Table 1:** Recoveries of the AZO sensor in tap-water samples.

Spiked AZO (nM)	Found AZO (nM)	Recovery (%)
15 ± 0.1	18 ± 1	119 ± 6
30 ± 0.2	29 ± 1	95 ± 4
50 ± 0.4	47 ± 3	94 ± 7

## Data Availability

Data is contained within the article and Supplementary Materials.

## Supplementary Materials

The following supporting information can be downloaded at: https://www.mdpi.com/article/10.3390/polym16101394/s1, Figure S1: The influence of DPV parameters: step size (a), pulse time (b) and pulse size (c) on the oxidation peak current of AZO sensor recorded in 0.3 M KCl solution containing 4 mM K_3_[Fe(CN)_6_]/K_4_[Fe(CN)_6_]. Each DPV underwent an individual baseline; Figure S2: A fragment of the first cycle of the cyclic voltammogram recorded on Au TFME in PBS at the presence of 5 mM Ani, 5 mM mPD, or mixture of 5 mM mPD and 5 mM Ani (see color codes on the graphs) at a scan rate of 100 mV/s; Figure S3: Attenuated total reflection (ATR) absorbance of AZO powder; Figure S4: CVs (a) and EIS (b) characterization of bare gold, poly(Ani-co-mPD)/AZO, AZOMIP and AZO on AZOMIP that are prepared on Au TFME. The measurements were performed in 0.3 M KCl solution containing 4 mM K_3_[Fe(CN)_6_]/K_4_[Fe(CN)_6_]; Figure S5: (a) Responses of AZO sensor based on AZO–MIPs formed from the different polymers upon incubation in 50 nM AZO solution in ultrapure water. (b) Responses of sensors modified with AZOMIP and NIP layers formed from poly(Ani-co-mPD); Figure S6: Structures of fungicides used for selectivity study; Scheme S1: AZO structure with highlighted oxygen atoms; Scheme S2: A schematic representation of possible binding interaction between Ani and mPD monomers with template AZO; Table S1: Binding energies (kcal/mol) of interactions between the functional monomers and AZO at O5 site as calculated by Gaussian’09 software; Table S2: Hydrogen bond binding energy of mPD and Ani towards different oxygen atoms of AZO as calculated by Gaussian’09 software; Table S3: Charge transfer resistance (Rct) values obtained from fitting EIS spectra to a Randles equivalent circuit consisting of a solution resistance (Rs), a constant phase element (CPE), a charge transfer resistance (Rct), and a Warburg impedance (Wd); Table S4: Selectivity coefficient (k) for rebinding of the fungicides to AZO sensor as calculated by Equation (SE1). References [53,54] are cited in the Supplementary Materials.
