# Supplementary material for: Molecularly Imprinted Polymer-Based Electrochemical Sensor for the Detection of Azoxystrobin in Aqueous Media
Source: Polymers (Basel). 2024 May 14;16(10):1394. doi: 10.3390/polym16101394 (PMC11125202; doi:10.3390/polym16101394)
Supplement: Supplementary file 1 [file polymers-16-01394-s001.zip › polymers-2964378-supplementary.pdf]

# Supplementary Information

# Molecularly Imprinted Polymer-Based Electrochemical Sensor for the Detection of Azoxystrobin in Aqueous Media

Vu Bao Chau Nguyen [1], Jekaterina Reut [1], Jörg Rappich [2], Karsten Hinrichs [3], and Vitali Syritski [1,*]

[1] Department of Materials and Environmental Technology, Tallinn University of Technology, Ehitajate tee 5, 19086 Tallinn, Estonia; vunguy@taltech.ee (V.B.C.N.)

[2] Young Investigator Group Nanoscale Solid-Liquid Interfaces, Helmholtz-Zentrum Berlin für Materialien und Energie GmbH, Schwarzschildstr. 8, 12489 Berlin, Germany; rappich@helmholtz-berlin.de

[3] Application Laboratories Berlin, Leibniz-Institut für Analytische Wissenschaften-ISAS-e.V., Schwarzschildstraße 8, 12489 Berlin, Germany; hinrichs@isas.de

* Correspondence: vitali.syritski@taltech.ee

## Table of contents

Table of contents ........ 1
S1. Optimization of Different Pulse Voltammetry parameters ........ 2
S2. Binding energy between AZO and the functional monomers ........ 2
S3. Electrochemical oxidation potential of ANI, mPD and AZO ........ 4
S4. Attenuated total reflection absorbance of AZO ........ 5
S5. Electrochemical characterization of poly(Ani-co-mPD) synthesis and AZO–MIP formation ........ 6
S6. Effect of polymers used to form AZO–MIPs on performance of AZO-sensor ........ 7
S7. Selectivity study of the AZO sensor ........ 8
References ........ 8

## S1. Optimization of Different Pulse Voltammetry parameters

Differential pulse voltammetry (DPV) parameters, such as step size (or potential step), pulse time (or pulse width), and pulse size (or pulse amplitude), were optimised to maximise the current response and precision while minimising the analysis time. Purpose to enhance sensitivity for accurately determining small relevant azoxystrobin concentrations in natural water.

Step size optimization was performed by recording DPV for various step size values while applying a pulse time of 100 ms and a pulse size of 25 mV (Figure S1a). The peak current increased with the growth of the step size, indicating a faster scan rate leading to a more rapid redox reaction and consequently, higher current responses [1,2]. Step sizes of 5, 7, and 9 mV were considered due to repeatable peak current values. The optimal step size of 7 mV was chosen to balance peak height, repeatability, and running time.

Pulse time optimization was performed by testing five values (20, 40, 60, 80, and 100 ms) using a pulse size of 25 mV and the previously chosen step size of 7 mV. Longer pulse widths resulted in lower peak current values as seen in Figure S1b. After considering peak height and repeatability of measurements, the optimal pulse time was determined to be 40 ms.

The pulse size was selected between 10 and 40 mV, while keeping the previously chosen step size and pulse time of 7 mV and 40 ms, respectively. Larger pulse size leads to greater electron transfer, resulting in increased peak currents (Figure S1c). The optimal value of 35 mV was chosen for better repeatability compared to others.

In short, optimization of DPV parameters aimed to balance peak height, repeatability, and running time for each parameter. The carefully selected parameter values - namely, a step size of 7 mV, a pulse time of 40 ms, and a pulse size of 35 mV - align with their respective impacts on scan rate, redox kinetics, and electron transfer processes.

## S2. Binding energy between AZO and the functional monomers

In this study Gaussian'09 software was used for the computational modelling allowing the assessment of hydrogen bonding between AZO and selected monomers including 2-methyl-4-nitroaniline (2M4N), 3-aminothiophenol (3ATP), aniline (Ani), meta-phenylenediamine (mPD), pyrazole (PRZ), and pyrrole (PYR). Since AZO molecule contains 5 oxygen atoms potentially capable of hydrogen bonding (Scheme S1), we initially focused on calculating the binding energy by linking the amino group hydrogen atom of each monomer to one oxygen atom (O5) of the AZO template (Table S1). Complex between AZO and Ani exhibited a higher binding energy followed by the complex AZO-mPD. These two monomers were selected for further studies. We performed calculations for the AZO-mPD and AZO-Ani complexes, where all geometrically possible binding sites between oxygen atoms of AZO and amino group hydrogen atoms of monomer were considered (Table S2). As it can be seen, the total binding energy of the complex of AZO with 5 Ani monomers is higher than that of the complex with 5 mPD monomers. A possible case of hydrogen interaction between the proton-donor groups of the monomers and the oxygen atoms of the template AZO was illustrated in Scheme S2.

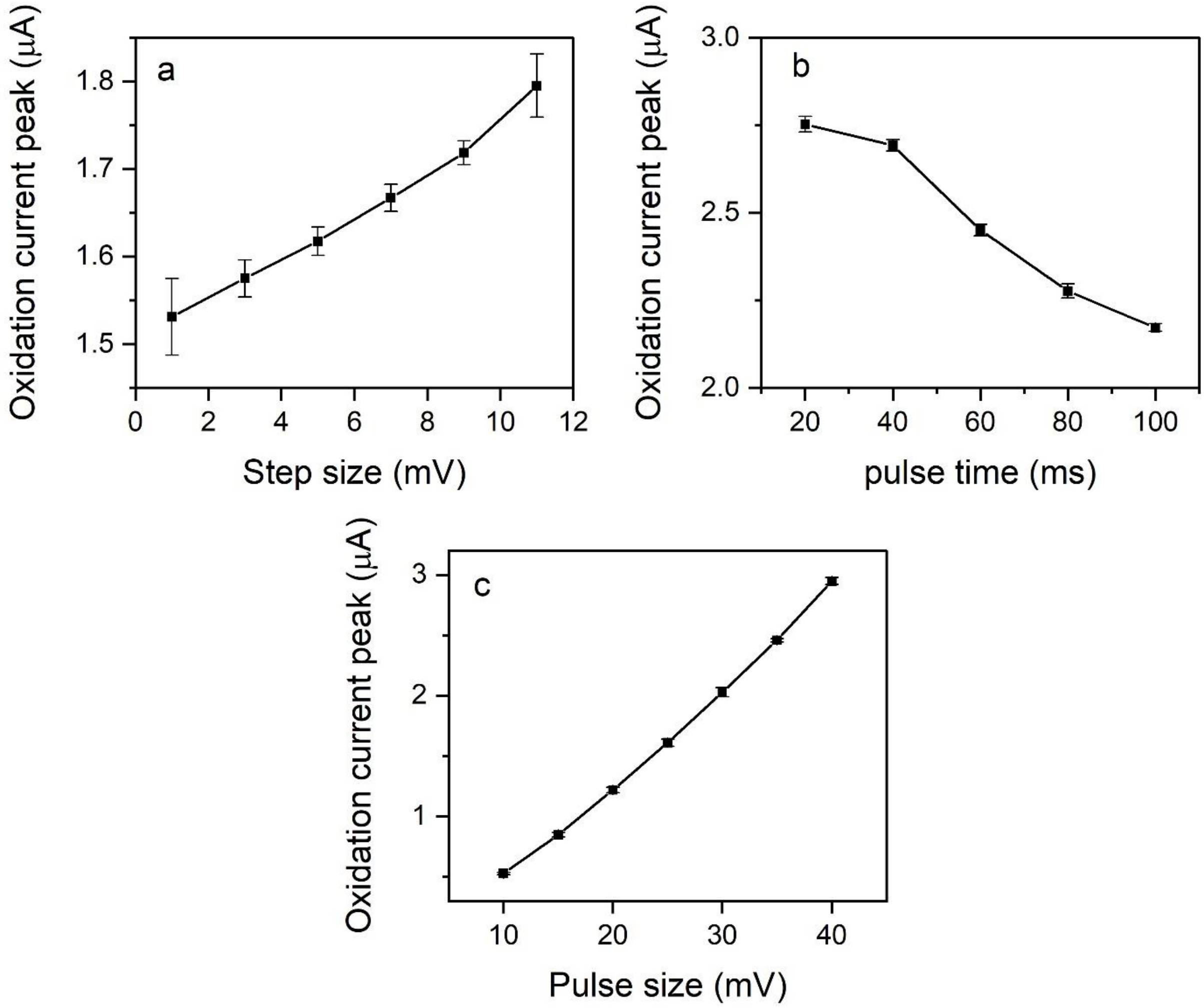


Figure S1. The influence of DPV parameters: step size (a), pulse time (b) and pulse size (c) on the oxidation peak current of AZO sensor recorded in 0.3 M KCl solution containing 4 mM $K_3[Fe(CN)_6]/K_4[Fe(CN)_6]$. Each DPV underwent an individual baseline.

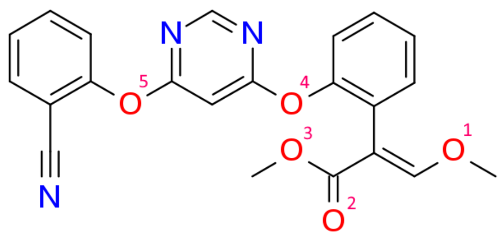

Scheme S1. AZO structure with highlighted oxygen atoms.

Table S1. Binding energies (kcal/mol) of interactions between the functional monomers and AZO at O5 site as calculated by Gaussian'09 software.

| **Monomer** | Ani | mPD | PRZ | PYR | 3-ATP | 2M4N |
|---|---|---|---|---|---|---|
| **Binding (kcal/mol)** | 103.684 | 89.279 | 87.148 | 86.471 | 85.948 | 83.860 |

Table S2. Hydrogen bond binding energy of mPD and Ani towards different oxygen atoms of AZO as calculated by Gaussian'09 software.

| Monomer | Binding energy (kcal/mol) | | | | | |
|---|---|---|---|---|---|---|
| | Oxygen (1) | Oxygen (2) | Oxygen (3) | Oxygen (4) | Oxygen (5) | **Total** |
| mPD | 144 | 149 | 163 | 135 | 89 | 680 |
| Ani | 187 | 206 | 165 | 205 | 104 | 867 |

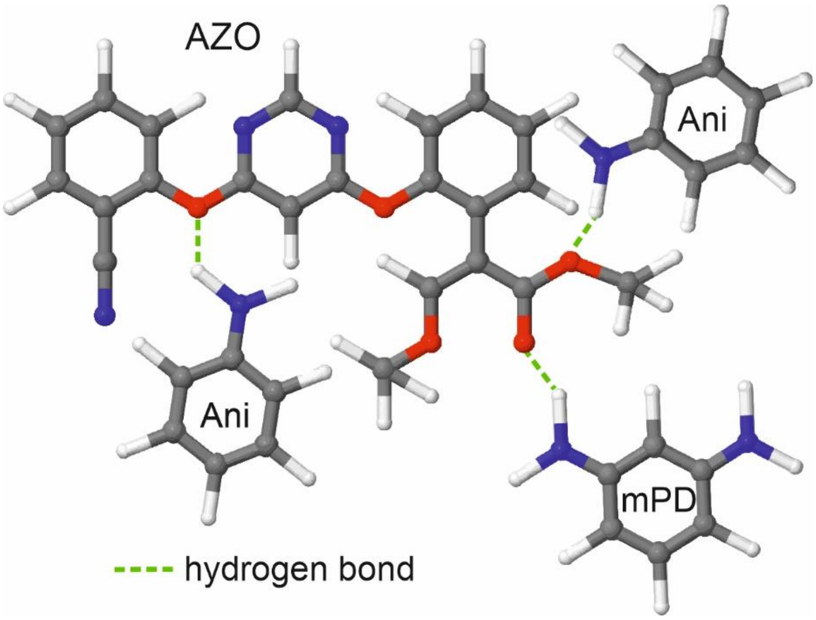


Scheme S2. A schematic representation of possible binding interactions between Ani and mPD monomers with template AZO.

## S3. Electrochemical oxidation potential of ANI, mPD and AZO

The oxidation of mPD and Ani start at about 250 mV and 300 mV, respectively. The oxidation of both monomers in their solution mixture, a potential starting from 300 mV is expected.

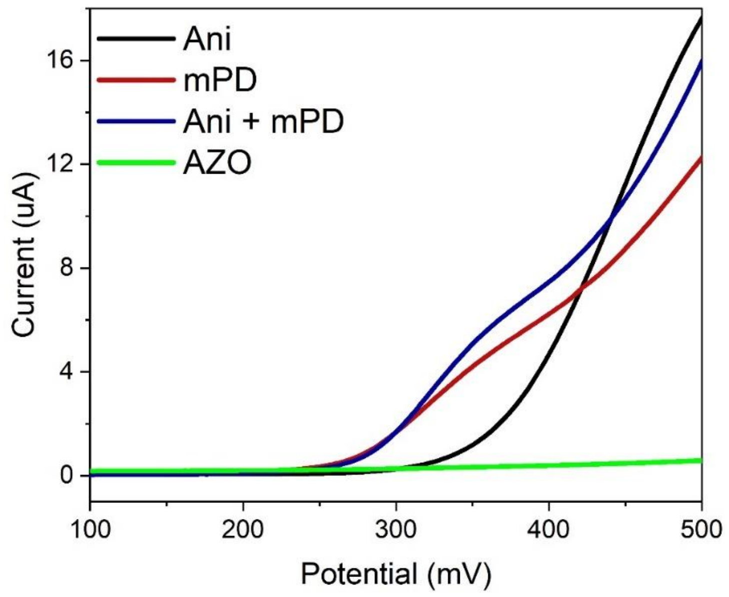


Figure S2. A fragment of the first cycle of the cyclic voltammogram recorded on Au TFME in PBS at the presence of 5 mM Ani, 5 mM mPD, or mixture of 5 mM mPD and 5 mM Ani (see colour codes on the graphs) at a scan rate of 100 mV/s.

**S4. Attenuated total reflection absorbance of AZO**

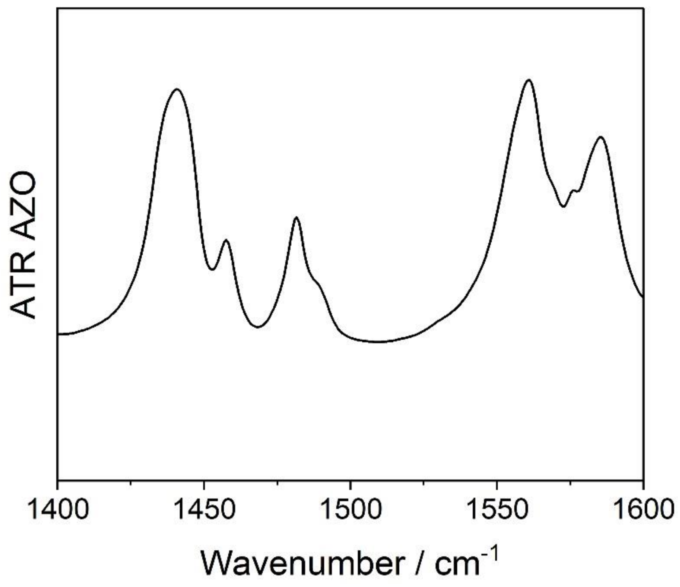


Figure S3. Attenuated total reflection (ATR) absorbance of AZO powder.

**S5. Electrochemical characterization of poly(Ani-co-mPD) synthesis and AZO–MIP formation**

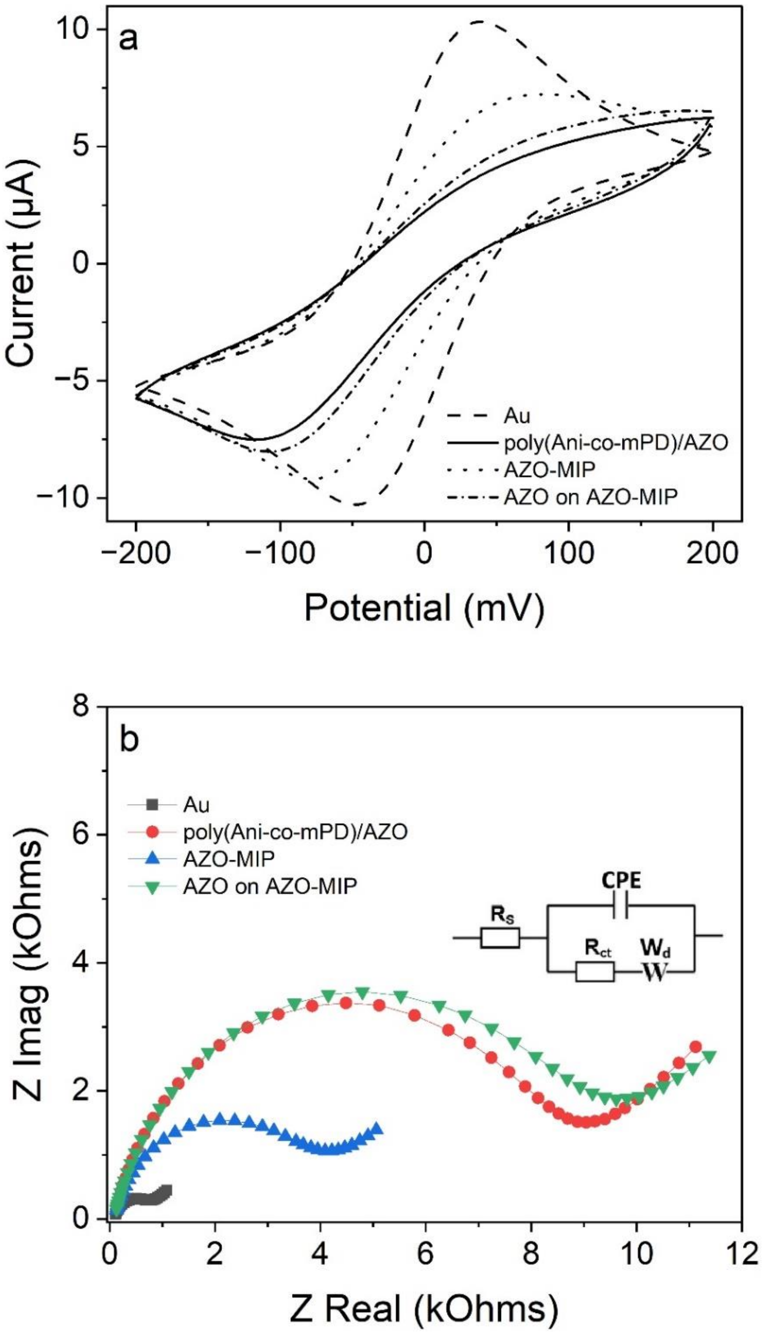


Figure S4. CVs (a) and EIS (b) characterization of bare gold, poly(Ani-co-mPD)/AZO, AZO–MIP and AZO on AZO–MIP that are prepared on Au TFME. The measurements were performed in 0.3 M KCl solution containing 4 mM $K_3[Fe(CN)_6]/K_4[Fe(CN)_6]$.

Table S3. Charge transfer resistance (Rct) values obtained from fitting EIS spectra to a Randles equivalent circuit consisting of a solution resistance (Rs), a constant phase element (CPE), a charge transfer resistance (Rct), and a Warburg impedance (Wd).

| Parameters | Au | poly(ANI-co-mPD)/AZO | MIP | AZO on MIP |
|---|---|---|---|---|
| Rct (kOhms) | 0.6 | 7.5 | 3.2 | 7.3 |

## S6. Effect of polymers used to form AZO–MIPs on performance of AZO-sensor

Three polymers, poly(Ani), poly(mPD) and poly(Ani-co-mPD) were use to prepare AZO–MIP layer on the surface of TFME and the responses of the AZO-sensors upon the incubation in 50 nM AZO solution in ultrapure water were recorded by DPV in the presence of a redox probe (Figure S5a). The AZO sensor with MIP layer synthesized from poly(Ani-co-mPD demonstrated the highest response.

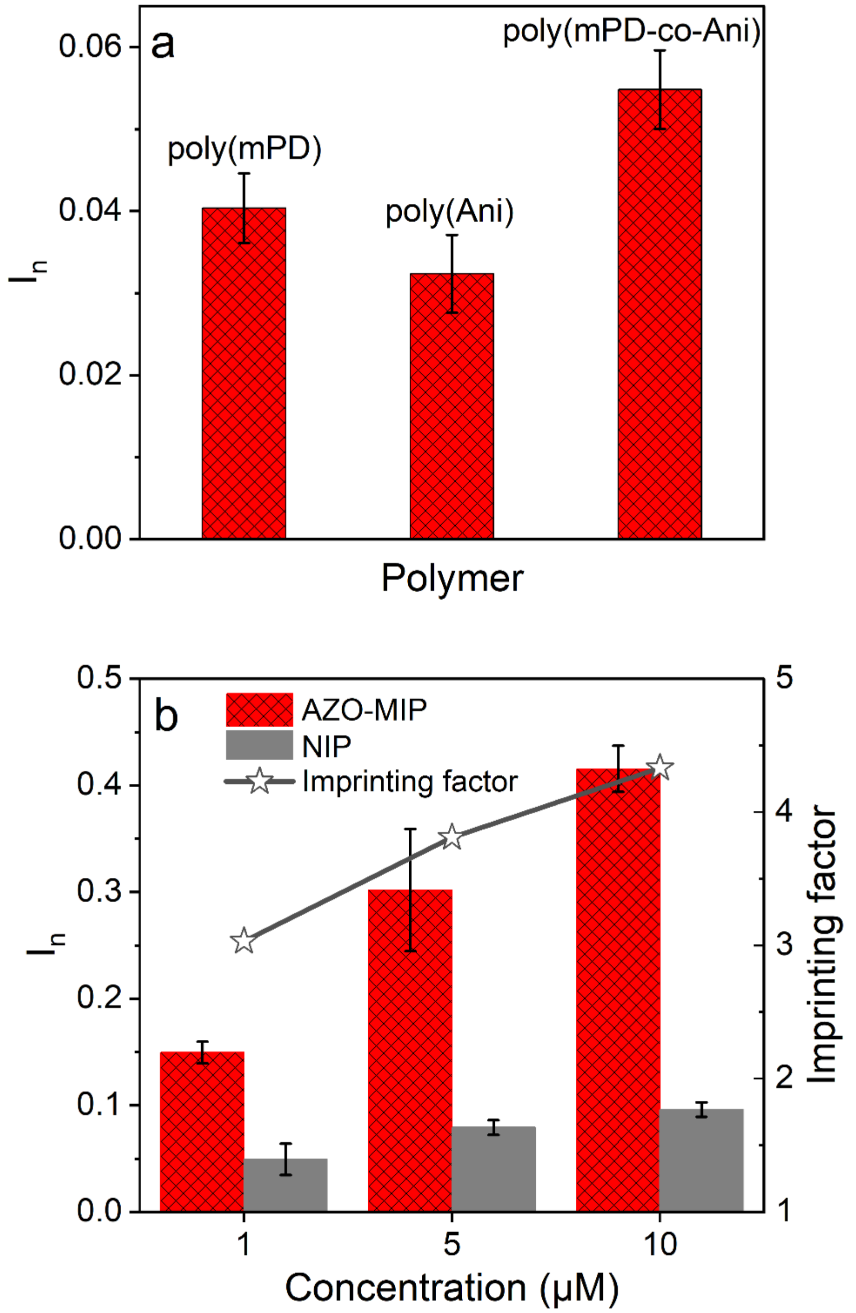


Figure S5. (a) Responses of AZO sensor based on AZO-MIPs formed from the different polymers upon incubation in 50 nM AZO solution in ultrapure water. (b) Responses of sensors modified with AZO–MIP and NIP layers formed from poly(Ani-co-mPD).

To characterize the success of the imprinting procedure the imprinting factor value was determined. For this purpose the responses of sensors modified with AZO-MIP and non-imprinted polymer (NIP) layers, both formed from poly(Ani-co-mPD), upon rebinding of three

different concentrations of AZO were measured. The IF value was calculated as the ratio of the response of AZO–MIP sensor to the response of NIP sensor (Figure S5b).

## S7. Selectivity study of the AZO sensor

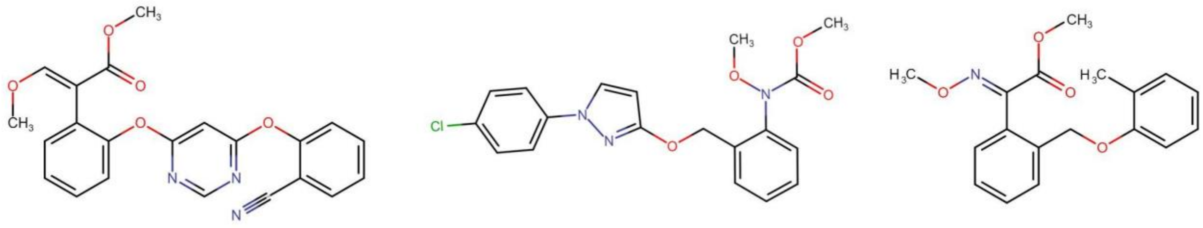


Azoxystrobin (AZO) 403.4 g/mol | Kresoxim-methyl (KSX) 313.3 g/mol | Pyraclostrobin (PRC) 387.8 g/mol

Figure S6. Structures of fungicides used for selectivity study.

The selectivity coefficient (k) was calculated by equation:

$k = I_n(\mathrm{t}) / I_n(\mathrm{i})$ (SE1)

where $I_n(\mathrm{t})$, $I_n(\mathrm{i})$ are the responses generated by the target (t) and interferent (i) molecules on the AZO–MIP.

Table S4. Selectivity coefficient (k) for rebinding of the fungicides to AZO sensor as calculated by Eq. SE1.

| | AZO | KSX | PRC |
|---|---|---|---|
| $I_n$(ultrapure water) | 0.037±0.002 | 0.018±0.002 | 0.009±0.003 |
| $I_n$(tap water) | 0.056±0.003 | 0.029±0.003 | 0.014±0.005 |
| k(ultrapure water) | - | 2.1 | 4.1 |
| k(tap water) | - | 1.9 | 4.0 |

## References

[1] M. Madej, J. Kochana, B. Baś, Determination of viloxazine by differential pulse voltammetry with boron-doped diamond electrode, Monatsh Chem 150 (2019) 1655–1665. https://doi.org/10.1007/s00706-019-2380-6.

[2] M. Gharous, L. Bounab, F.J. Pereira, M. Choukairi, R. López, A.J. Aller, Electrochemical Kinetics and Detection of Paracetamol by Stevensite-Modified Carbon Paste Electrode in Biological Fluids and Pharmaceutical Formulations, IJMS 24 (2023) 11269. https://doi.org/10.3390/ijms241411269.
